# Quantifying antimicrobial use on Canadian dairy farms using garbage can audits

**DOI:** 10.3389/fvets.2023.1185628

**Published:** 2023-06-30

**Authors:** Landon M. C. Warder, Luke C. Heider, David F. Léger, Daniella Rizzo, J. T. McClure, Ellen de Jong, Kayley D. McCubbin, Tamaki Uyama, Mariana Fonseca, Ana Soffia Jaramillo, David F. Kelton, David Renaud, Herman W. Barkema, Simon Dufour, Jean-Philip Roy, Javier Sánchez

**Affiliations:** ^1^Department of Health Management, Atlantic Veterinary College, University of Prince Edward Island, Charlottetown, PE, Canada; ^2^Public Health Agency of Canada, Guelph, ON, Canada; ^3^Faculty of Veterinary Medicine, University of Calgary, Calgary, AB, Canada; ^4^Department of Population Medicine, Ontario Veterinary College, University of Guelph, Guelph, ON, Canada; ^5^Faculty of Veterinary Medicine, Université de Montréal, Montréal, QC, Canada

**Keywords:** antimicrobial use (AMU), dairy, garbage can audit, antimicrobial stewardship (AMS), meta-analysis, temporal change, Canada

## Abstract

Antimicrobial resistance in pathogenic bacteria is one of the preeminent concerns for the future of global health. There is a dose-dependent relationship between antimicrobial use (AMU) and the prevalence of antimicrobial-resistant pathogens. As most AMU in Canada is related to animal agriculture, there is a need to reduce overall AMU, which could be accomplished through surveillance of AMU in animal agriculture, including the dairy industry. The objective of this study was to quantify AMU on dairy farms across Canada. This study had two parts: a description of data collected in 2019–2020, and a meta-analysis comparing this data to previous estimates of AMU in the Canadian dairy industry. The first included a garbage can audit (GCA) on 107 farms in four Canadian provinces (British Columbia, Alberta, Ontario, and Nova Scotia) in 2020; AMU data were converted to the dose-based metrics of defined course doses (DCD) and defined daily doses (DDD). Mixed-effect linear models were fit to determine the relationship between province and use of different classes of antimicrobials. On average, for every 100 animals on the farm, 117 DCD of antimicrobials were administered per year (IQR: 55, 158). These treatments amounted to 623 DDD / 100 animal-yr (IQR: 302, 677 DDD/100 animal-years). Penicillins were the most used class of antimicrobials, followed by first-and third-generation cephalosporins. Farms in Ontario used more third-generation cephalosporins than other provinces. The second part of this study compared AMU in 2020 to previously reported Canadian studies through a meta-analysis. A GCA was conducted in 2007–2008 in Alberta, Ontario, Québec, and the Maritime provinces (Prince Edward Island, New Brunswick and Nova Scotia); another GCA was conducted in Québec in 2018. Overall, AMU was lower in 2018–2020 than in 2007–2008, with the exception of third-generation cephalosporin use, which increased.

## 1. Introduction

Antimicrobial resistance (AMR) is one of the most significant threats to global health (1). The Government of Canada has identified antimicrobial use (AMU) in animals as a target to reduce the development of resistance in zoonotic, pathogenic bacteria (2). To promote this, the Canadian government has encouraged prudent AMU in livestock and increased antimicrobial oversight of pharmaceuticals for veterinary use (Government of Canada, 2019). Addressing AMR in the Canadian dairy industry requires expressing and comparing AMU within the industry and across countries and industries (3, 4) to develop benchmarks for assessing future interventions.

Farm-level AMU information can be retrieved using different data sources (5). For instance, it can be obtained using treatment records, the most straightforward source of AMU because they directly measure the amount used (6). However, it has been observed that dairy farmers do not keep complete records of all the treatments, and therefore they underestimate AMUs (7–10).

Another approach to estimating AMU is the garbage can audit (GCA). This method consists of placing a receptacle in convenient locations on a farm, and farmworkers are instructed to discard all drug containers into the receptacle for a defined period (10). Discarded containers are then counted to measure the disappearance rate of antimicrobials on the farm and to estimate AMU. A GCA tends to correlate well with AMU quantified through veterinary dispensing records, but may report somewhat lower usage (11). The main advantage of a GCA is that it is simple and requires little logistical development compared to other methods. They also circumvent issues derived from farms obtaining antimicrobials from multiple vendors.

A GCA can give the amount of products used, but converting these to dose-based metrics (DBM) is useful so that AMU can be compared regarding therapeutic potential. Conversion factors are established so that a specific mass confers a single course of treatment or day of effect. Conversion factors must be calculated for each active ingredient (AI) for a given route of administration [ESVAC (12)]. Lardé et al. (13) computed the defined daily dose (DDD) and defined course dose (DCD) conversion factors for every antimicrobial approved for use in cattle in Canada, specified as DDD_bovCA_ and DCD_bovCA_ (13).

To account for variation in farm size and duration of observation, an antimicrobial drug use rate (ADUR) is used to express the DBM in terms of the amount of animal time exposed (10). ADURs can be computed for any type of DBM denoted by a subscript, such as ADUR_DDD_ or ADUR_DCD_.

The objective of this study was to assess AMU in Canadian dairy farms. Firstly the relationship between AMU, management practices and farm characteristics will be summarized. Then, a meta-analysis was used to compare AMU between 2007–2008 and 2017–2020, using the GCA methodology.

## 2. Materials and methods

### 2.1. Data sources

Three GCA studies were used for this study. Two have been previously reported in the literature (10, 11). Saini et al. (10) provided the baseline while Lardé et al. (11) and the current study, as outlined in Fonseca et al. (14), provided a follow up. Many of the farms were the same in the baseline and follow-ups. The inclusion and exclusion criteria were very similar in all three studies and all aimed to be representative of commercial dairy farms in each province assessed.

The first study was from 2007 to 2008, when a GCA was conducted on 89 dairy farms in Alberta, Ontario, Québec, and the Maritime provinces of Canada (10). The average herd size was 84 cows with an average per cow daily milk production was 32 kg. The median SCC was 220,000 cells/mL and 61% used tie-stalls. For further information, refer to Saini et al. (10). Raw count data from the 2007 to 2008 GCA were obtained and processed using the same methods for all GCAs.

The second study was conducted in 2017–2018 on 101 dairy farms in Québec as described by Lardé et al. (11). The mean herd had 67 cows with a per cow butterfat production of 1.2 kg/day. The garbage cans were left on the farm for over 1 year. Data for comparison to the 2019–2020 GCA were retrieved from the Supplementary material of Lardé et al. (11).

The third study was conducted in 2019–2020 on 106 dairy farms in British Columbia, Alberta, Ontario, and Nova Scotia (14). Farms were a convenience sample recruited through their local veterinarian, and receptacles were placed in convenient locations on the farm.

The average herd size was 135 cows with a daily milk and butterfat output of 35 kg/cow and 1.4 kg/cow. The median somatic cell count (SCC) was 175,000 cells/mL. 13% of farms used tie stalls for their lactating herd. Tie-stalls are more common on smaller herds and in Québec, so this proportion is close to what would be expected of commercial dairy farms in the studied regions (15). The Code of Practice has been encouraging the reduction of tie stalls in Canada, so a marked reduction in their popularity is to be expected (16). Farm personnel were asked to discard all medications into the receptacle, whether they had an antimicrobial activity or not. At the start of the observation period, a farm pharmacy inventory was conducted to determine what antimicrobials the farms already had in stock. At this time, a questionnaire was completed to gather herd demographic information. Three times over the next six to 12 months, the garbage can was emptied, and the contents were counted. A second pharmacy inventory was conducted at the last visit. For more details, refer to Fonseca et al. (14).

### 2.2. Data base metrics

**Figure 1:**
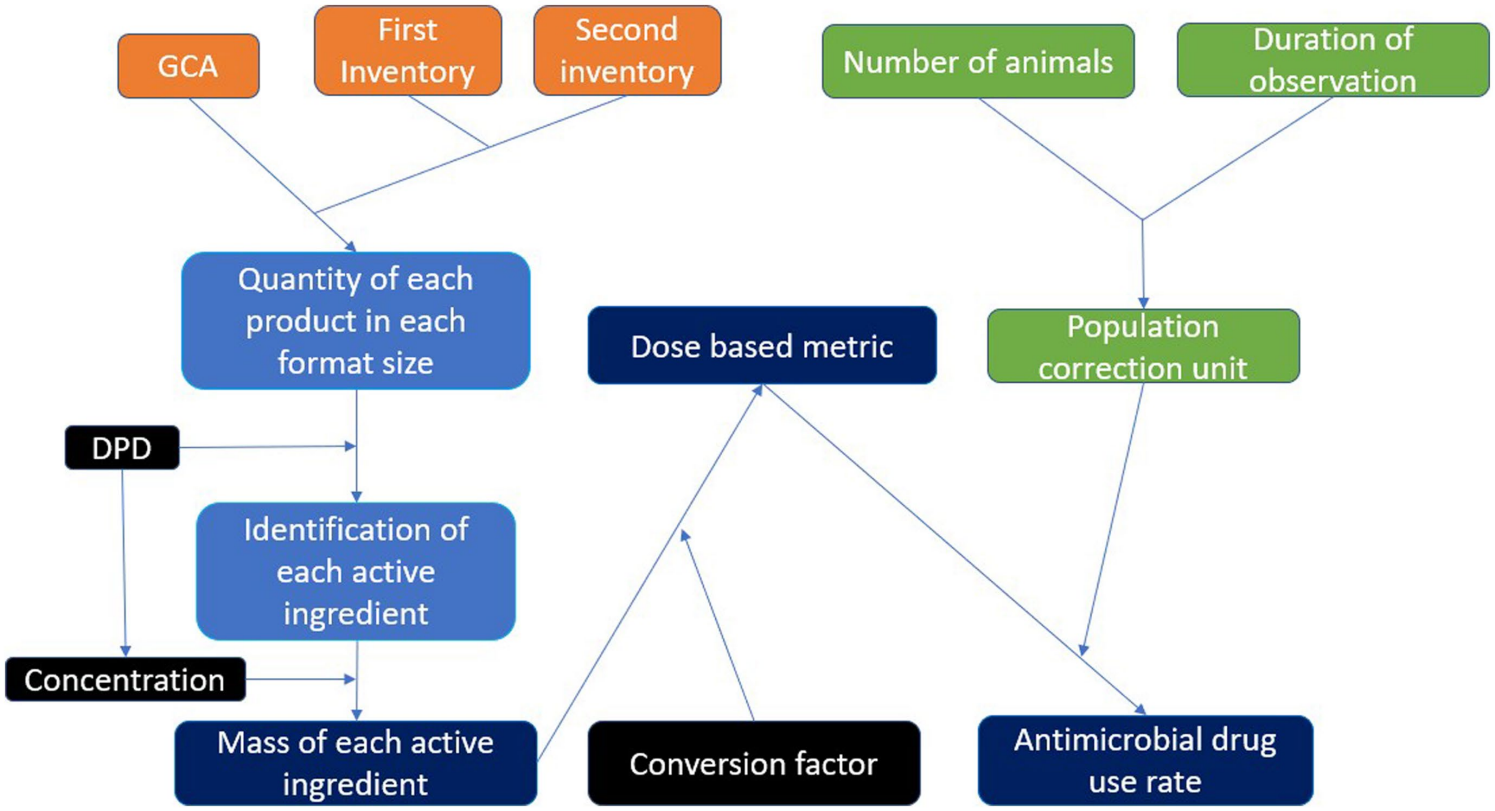
A flow diagram to demonstrate the flow of data from the garbage can audit to dose based metrics. Orange represents primary AMU data, green represents herd metadata, black represents external reference data, light blue represents data processing steps, and dark blue represents AMU metrics.

All data processing involved in computing DBMs was done using Python 3.9 in the integrated development environment PyCharm 2021.2 (JetBrains s.r.o, Prague, Czechia). A program was written to convert the GCA data into a standardized format. A diagram of the flow of data is presented in Figure 1.

The equation used to convert the number of drug containers found into the DBM of a single active ingredient in a product is expressed as:


$$\begin{array}{l} DBM_{AI\cap Product\cap Format}=\\ \;\;\;\;\;\;\;\;\;\;\;\;\;\frac{N_{Product\cap Format}.\;\;Format\;.\;\;Conc\;.{\;}_{AI\cap Product}}{ConversionFactor_{DBM\cap AI\cap ROA}} \end{array}$$

Where the DBM for an active ingredient (AI) in a product for a given product size (format) can be expressed in terms of either DCD or DDD. $N_{Product\cap Format}$ is the number of items of a given product in a particular size. The format is the size of the product. The concentration is specific to each active ingredient in the product and taken from Health Canada’s drug product database. The product of these three variables equals the mass of the active ingredient. The mass is then converted to a DBM, be it DCD or DDD, with a conversion factor that is specific for an active ingredient and route of administration (13).

These metrics allow for fair product comparisons, but, in order to compare farms, the metrics should be corrected by the number of animals and time between the initial and end visits (duration). This can be calculated for either DDDs or DCDs using the equation:


$$ADUR_{DBM}=\frac{\sum DBM_{AI\cap Product\cap Format}}{N_{animals}\;.\;\;Duration}$$

Where the ADUR for a farm is the sum of DDDs or DCDs for every AI used on the farm during the observation period, divided by the number of animals and the amount of time for which the animals were exposed. This equation gives results in the number of courses of treatment (DCD) or the number of days of effect of those courses (DDD) per 100 animal-years. 100 animals-years was chosen to indicate AMU in terms of a 100-animal herd for a year.

### 2.3. Statistical analyses

Dose-based metrics were also expressed for chemical class and each category of importance. The Health Canada drug product database was used to determine both the chemical class of the antimicrobial and its category of importance (17). The chemical class refers to the chemical family of antimicrobial, such as penicillin or tetracycline. Health Canada ascribed a category of importance, from 1 (very high importance) to 4 (low importance), to each class of antimicrobials to indicate their importance to human medicine. Only Categories 1, 2, and 3 are considered medically important.

Statistical analyses were done in STATA16 (StataCorp., College Station, Texas, United States). Averages and interquartile ranges (IQR) were computed from the ADUR_DDD_ and ADUR_DCD_. AMU was stratified by route of administration, category of importance, and the chemical class of the active ingredient. All statistical models were assessed for homoscedasticity with the Breusch-Pagan/Cook-Weisberg method. Deletion residuals were used to assess models of third-generation cephalosporins, for normality and outliers. Standardized residuals and BLUPs were used to assess models of all chemical classes.

#### 2.3.1. AMU and herd-level characteristics

For the 2019–2020 GCA, data were obtained from each farm regarding cow-level somatic cell count (SCC), milk production per 305-day lactation, and herd size through their dairy herd improvement program. The SCC and 305-day milk production were averaged for all cows lactating at the sampling time closest to January 1, 2020, which was the approximate middle of the observation period. The herd size included animals in all production phases.

Linear regression models were assessed to evaluate the relationship between each of these three variables and the total ADUR_DDD_, as well as the ADUR_DDD_ of third-generation cephalosporins. Separate multivariable mixed-effect linear regression models for total ADUR_DDD_ were built with SCC, milk production, and herd size, including a term for the chemical class of antimicrobial, and clustered on the farm. The relationship between AMU and third-generation cephalosporins used a simple, univariable linear regression for each of the three herd metrics. The total and third-generation cephalosporin ADUR_DDD_ was log-transformed to fit model assumptions. These models do not infer causality, so each association was considered to be of interest individually.

#### 2.3.2. Comparison of AMU in 2007–2008 to 2017–2020

Modeling with all three data sets investigated how the data from 2007–2008 differed from that of the more recent studies, completed in 2017–2018 and 2019–2020. All three studies had the same target population of Canadian commercial dairy farms to provide surveillance of antimicrobial use at each time point. This provided follow-ups for Alberta, Ontario, Québec, and the Maritime provinces. Many farms were included in the original study and the follow ups, but were kept anonymized because of data privacy concerns. The 2007–2008 study only recorded the number of dry and lactating animals on the farm, so all ADUR_DDD_s used the population correction unit of 100 cow-years. Only parenteral use was assessed due to differences in how studies approached medicated feed. There are no medically important antimicrobials approved for feed in lactating dairy cows in Canada (11).

Two sets of models were built to assess the shifts in AMU between the study by Saini et al. (10) and the later studies. Each province was modeled separately to determine how pervasive observed associations were. When significance was evaluated, α was set at 0.05. All assessments were determined *a priori.* A mixed-effects linear model was built to predict the amount of each class of antimicrobial that was used on a farm, given that it was above zero. Predictors were the time point, the chemical class of the antimicrobial, and the interaction between the two. Models were clustered on the farm.

## 3. Results

The most recent study, conducted in 2019–2020, collected data from 106 farms across British Columbia, Alberta, Ontario, and Québec. The ADUR_DDD_ was 623 DDD/100 animal-yr (IQR: 302, 677), and the ADUR_DCD_ was 117 DCD/100 animal-yr (IQR: 55, 158). Table 1 displays the ADUR_DDD_ and ADUR_DCD_ for each category of importance. One farm used a partial bag of chlortetracycline premix, which accounted for more than 10% of all of the DDDs in the study. Exclusion of this farm resulted in the ADUR_DDD_ of 514 DDD/100 animal-yr (IQR: 302, 666), and the ADUR_DCD_ was 115 DCD/100 animal-yr (IQR: 55, 157), a reduction of 17 and 1.7%, respectively.

**Table 1:** Mean and interquartile range ADUR_DDD_ and ADUR_DCD_ by category of importance to human medicine in DDD/100 animal-years and DCD/100 animal-years for the CaDNetASR project (2019–2020).

	ADUR_DDD_	ADUR_DCD_
Category I	115 (28, 129)	31 (11, 39)
Category II	333 (170, 468)	65 (28, 88)
Category III	138 (1, 32)	9 (0, 10)
Total	623 (302, 677)	117 (55, 158)

### 3.1. AMU by chemical class in 2019–2020

Fluoroquinolones and third-generation cephalosporins are the two classes of Category I antimicrobial active ingredients available for use in Canadian dairy cattle. Fluoroquinolones were rarely used on study farms (*n* = 11), with a maximum ADUR_DCD_ of 7 DCD per 100 animal-yr. Ceftiofur, a third-generation cephalosporin, was the most used Category I antimicrobial, and it was used on all but five (95%) farms in the 2019–2020 study. The ADUR_DCD_ was 22 DCD/100 animal-yr (IQR: 5, 28) and the ADUR_DDD_ was 105 (IQR: 19, 110).

The most used chemical class of antimicrobial was penicillins, which are Category II. Penicillins accounted for 163 DDD/100 animal-years and 31 DCD/100 animal-yr, which is 26% of total use for both metrics.

### 3.2. AMU by route of administration in 2019–2020

Every farm used at least one product labeled for systemic administration. Systemic administration accounted for 31% of total usage, ranging from 0.6% to 100%. One hundred and one farms (95%) used intramammary (IMM) antimicrobials. IMM use contributed 66% of total AMU, ranging from 0% to 99%. Only 33 farms used products designated for oral, topical, or intrauterine use, averaging 3% of usage, ranging from 0% to 94%.

By ADUR_DDD_, first-generation cephalosporins were the most used IMM antimicrobials (111 DDD/100 animal-yr). Penicillins were used at a similar level (105 DDD/100 animal-yr), with third-generation cephalosporins (70 DDD/100 animal-yr) and lincosamides (4 DDD/100 animal-yr) being used less commonly. By ADUR_DCD_, penicillins were the most used at 19 DCD/100 animal-yr (IQR: 0.2, 34). This was followed by third-generation cephalosporins (15 DCD/100 animal-yr), first-generation cephalosporins (14 DCD/100 animal-yr), and lincosamides (0.7 DCD/100 animal-yr).

With respect to third-generation cephalosporins, most of their usage was due to intramammary products. Out of the 105 days/100 animal-years (IQR: 16, 92) of third-generation cephalosporins by all ROAs, 70 (IQR: 0, 43) were given by the intramammary route. Similarly, out of the 22 courses/100 animal-year total, 15 (IQR: 0, 15) were from intramammary products.

The preferred IMM antimicrobial differed by province. Ontario farms used the most third-generation cephalosporin IMM products, whereas Alberta and Nova Scotia farms had higher ADUR_DCD_ for penicillins. With respect to British Columbia, no difference was found in IMM treatment between third-generation cephalosporins and penicillins.

### 3.3. AMU by region in 2019–2020

Overall, linear regression showed that AMU differed between regions (ADUR_DDD_: *p* = 0.015; ADUR_DCD_: *p* = 0.011) (Supplementary material). There was insufficient evidence that systemic use of antimicrobials differed significantly across regions (ADUR_DDD_: *p* = 0.28; ADUR_DCD_: *p* = 0.22). Intramammary use differed significantly by ADUR_DCD_ (*p* = 0.019), though ADUR_DDD_ did not reach significance (*p* = 0.15). Usage in each province, by ADUR_DDD_ and ADUR_DCD_ for both of these routes, as well as overall use, can be seen in Tables 2, 3_,_ respectively.

**Table 2:** The ADUR_DDD_ for each region is presented for all parenteral use, as well as for systemic use and intramammary use.

	Systemic		Intramammary		Total	
	Mean	IQR	Mean	IQR	Mean	IQR
British Columbia	112	73, 118	231	110, 336	343	231, 407
Alberta	153	75, 148	364	237, 518	518	302, 670
Ontario	197	71, 186	434	391, 551	633	434, 733
Nova Scotia	117	33, 144	365	199, 503	482	344, 607

**Table 3:** The ADUR_DCD_ for each region is presented for all parenteral use, as well as for systemic use and intramammary use.

	Systemic		Intramammary		Total	
	Mean	IQR	Mean	IQR	Mean	IQR
British Columbia	25	16, 26	44	18, 52	69	41, 71
Alberta	34	17, 36	104	50, 154	138	72, 190
Ontario	41	18, 39	80	43, 111	123	60, 174
Nova Scotia	24	8, 32	92	42, 117	117	66, 147

### 3.4. Association between AMU and herd-averaged cow-level metrics in 2019–2020

Herd size was significantly (*p* < 0.001) and negatively correlated (*r* = −0.17) with the total ADUR_DDD._ Average 305-day milk production (*p* = 0.678) and SCC (*p* = 0.969) did not demonstrate a significant relationship with total ADUR_DDD_ (Supplementary material). The ADUR_DDD_ of third-generation cephalosporins was significantly associated with SCC (*p* = 0.025) and 305-day milk production (*p* = 0.029), but not herd size (*p* = 0.177). SCC (*r* = −0.24) was negatively correlated with the ADUR_DDD_ of third-generation cephalosporins, while milk production (*r* = 0.23) was positively associated. Tables 4, 5 describe the results of these models for the overall ADUR_DDD_ and third-generation cephalosporins ADUR_DDD_.

**Table 4:** Herd-level factors associated with the natural logarithm of the ADUR_DDD_ for each class of antimicrobial after mixed-effects linear regression clustered on farm.

Variable	Coefficient	*p*-value	95% CI
Herd size (100 animals)	−0.101	**0.001**	−0.159, −0.044
SCC (1,000 Cells)	0.053	0.795	−0.350, 0.457
Milk per lactation (1,000 L)	0.028	0.606	−0.079, 0.136

Bolded values are significant at 0.05.

**Table 5:** Herd-level factors associated with the natural logarithm of the ADUR_DDD_ for third-generation cephalosporins after linear regression.

Variable	Coefficient	*p*-value	95% CI
Herd size (100 animals)	−0.082	0.177	−0.201, 0.038
SCC (1,000 Cells)	−0.879	**0.025**	−1.647, −0.111
Milk per lactation (1,000 L)	0.232	**0.029**	0.025, 0.440

Bolded values are significant at 0.05.

### 3.5. Comparison of AMU between 2007–2008 and 2019–2020

There were changes in AMU between the study by Saini et al. (10) and those conducted by Lardé et al. (11) and Fonseca et al. (14). Total AMU decreased from 284 to 161 DCD/100 cow-years (*p* < 0.001) as assessed through univariable mixed effect linear regression. There was a shift from polymyxin use to third-generation cephalosporins among Category I antimicrobials, likely attributable to the introduction of IMM ceftiofur in 2008 and the withdrawal of polymyxin-containing products from the Canadian market in 2020. New regulations regarding Category I antimicrobials came into effect in Québec in 2019, which Millar et al. (18) showed caused a profound reduction in the use of these products. However, this occurred after this study’s second assessment of AMU in Québec. Tables 6, 7 show the ADUR_DCD_ and ADUR_DDD_, respectively, for each chemical class of antimicrobial at both time points. Beyond the amount used, the number of farms using each chemical class changed. Table 8 demonstrates the number and percentage of farms at each time point using at least one product containing each chemical class of antimicrobial.

**Table 6:** The interquartile range and average parenteral ADUR_DCD_ (DCD/100 adult cow-years) of farms by class and timepoint, as well as the number of herds that used the products containing the drug class.

	2007–2008			2017–2020		
Drug class	Herds (%)	ADUR_DCD_	IQR	Herds (%)	ADUR_DCD_	IQR
3rd generation cephalosporins	80 (90)	12.7 (8.2)	3.27, 19.2	190 (92)	30.7 (14.9)	5.00, 36.1
Polymyxins	84 (94)	51.2 (38.4)	12.5, 66.7	144 (70)	23.7 (11.0)	0, 38.0
Fluoroquinolones	4 (4.5)	0.24 (0)	0, 0	13 (6.3)	0.35 (0)	0, 0
Macrolides	27 (30)	6.75 (0)	0, 2.98	61 (30)	5.43 (0)	0, 2.49
TMS	68 (76)	8.61 (4.78)	0.63, 11.5	161 (78)	6.17 (3.41)	0.72, 8.77
Penicillins	88 (99)	122 (112)	66.1, 152	200 (0.97)	62.2 (50.7)	24.7, 85.7
Lincosamides	50 (58)	5.39 (0.33)	0, 5.42	96 (47)	4.14 (0)	0, 4.71
Aminoglycosides	84 (94)	57.3 (42.7)	13.9, 74.1	149 (72)	26.2 (11.7)	0, 42.6
1st generation cephalosporins	76 (85)	34.8 (15.4)	3.80, 54.4	129 (63)	26.7 (4.96)	0, 31.9
Amphenicols	29 (33)	1.72 (0)	0, 1.91	84 (41)	3.98 (0)	0, 4.14
Tetracyclines	56 (63)	15.8 (6.13)	0, 17.8	96 (47)	9.95 (0)	0, 9.68
Sulfonamides	0 (0)	N/A	0, 0	20 (9.7)	2.21 (0)	0, 0
Overall	89 (100)	400 (327)	212, 504	206 (100)	231 (173)	118, 287

**Table 7:** The interquartile range and average parenteral ADUR_DDD_ (DDD/100 adult cow-years) of farms by class and timepoint, as well as the number of herds that used the products containing the drug class.

	2007–2008			2018–2020		
Drug class	Herds (%)	ADUR_DDD_	IQR	Herds (%)	ADUR_DDD_	IQR
3rd generation cephalosporins	80 (90)	56.6 (36.5)	15.1, 89.5	190 (92)	140 (57.4)	15.8, 139.3
Polymyxins	84 (94)	51.2 (38.4)	12.5, 66.7	144 (70)	23.7 (11.0)	0, 38.0
Fluoroquinolones	4 (4.5)	0.79 (0)	0, 0	13 (6.3)	1.16 (0)	0, 0
Macrolides	27 (30)	45.6 (0)	0, 12.8	61 (30)	37.0 (0)	0, 17.0
TMS	68 (76)	34.4 (19.1)	2.52, 46.0	161 (78)	24.7 (13.7)	2.88, 35.1
Penicillins	88 (99)	621 (660)	369, 828	200 (0.97)	305 (253)	75.3, 456
Lincosamides	50 (58)	27.0 (1.63)	0, 27.1	96 (47)	20.7 (0)	0, 23.5
Aminoglycosides	84 (94)	85.7 (64.0)	20.8, 111	149 (72)	40.9 (18.6)	0, 66.2
1st generation cephalosporins	76 (85)	135 (33.8)	6.50, 153	129 (63)	143 (6.24)	0, 216
Amphenicols	29 (33)	6.88 (0)	0, 7.63	84 (41)	15.93 (0)	0, 16.6
Tetracyclines	56 (63)	43.9 (17.1)	0, 49.5	96 (47)	14.9 (0)	0, 21.1
Sulfonamides	0 (0)	N/A (0)	0, 0	20 (9.7)	0.40 (0)	0, 0
Overall	89 (100)	1,470 (1278)	909, 1873	206 (100)	876 (835)	528, 1,103

**Table 8:** The percentage of farms in each province that used each chemical class of antimicrobial at each time point.

	2007–2008				2018–2020			
	Alberta	Nova Scotia	Ontario	Quebec	Alberta	Nova Scotia	Ontario	Quebec
Aminoglycosides	100	94	89	96	87	75	48	80
Amphenicols	53	24	33	25	70	25	68	23
1st generation cephalosporins	88	94	78	86	50	65	71	66
Fluoroquinolones	12	0	0	7	17	5	6	1
Lincosamides	71	41	59	61	20	29	19	75
Macrolides	29	18	33	36	33	20	29	27
Penicillins	100	100	96	100	100	100	90	100
Polymyxins	100	94	89	96	87	88	52	79
Sulfonamides	0	0	0	0	10	5	6	11
TMS	88	71	63	86	77	88	89	78
Tetracyclines	88	35	56	71	57	30	45	49
3rd generation cephalosporins	88	94	100	79	97	85	90	93

As seen in Table 9, though there was a drop in both systemic and intramammary use, the drop in intramammary use was far greater than in systemic use. Table 10 shows that the change in the use of intramammary antimicrobials. The drop in IMM administration is largest in penicillins, while the use of third-generation cephalosporin increased dramatically, due to the introduction of IMM ceftiofur products in 2008.

**Table 9:** The mean parenteral ADUR_DCD_ (DCD/100 adult cow-years) and ADUR_DDD_ (DDD/100 adult cow-years) of systemic and intramammary use are presented for the two timepoint observed, as well as the number of herds that used any product through the given route of administration.

	2007–2008			2018–2020		
Route	Herds (%)	ADUR_DCD_	ADUR_DDD_	Herds (%)	ADUR_DCD_	ADUR_DDD_
Systemic	88 (99)	31	135	204 (99)	25	114
Intramammary	87 (98)	336	1,200	201 (98)	172	647

**Table 10:** The mean parenteral ADUR_DCD_ (DCD/100 adult cow-years) and ADUR_DDD_ (DDD/100 adult cow-years) of intramammary antimicrobials for the four main chemical classes for the route of administration, as well as the number of farms that used each chemical class in an intramammary product.

	2007–2008			2018–2020		
Drug name	Herds (%)	ADUR_DCD_	ADUR_DDD_	Herds (%)	ADUR_DCD_	ADUR_DDD_
First generation cephalosporins	73 (82)	34	135	107 (52)	25	141
Third generation cephalosporins	28 (31)	2	4	146 (71)	21	92
Lincosamides	52 (58)	5	27	96 (47)	4	21
Penicillins	87 (98)	103	537	173 (84)	43	218

A drop in ADUR_DCD_ and ADUR_DDD_ was observed in each region observed at both time points. Table 11 shows the ADUR_DCD_ and ADUR_DDD_ for each region at each time point. British Columbia was only evaluated in 2019–2020, but had lower use than any other region at that time. In multivariable mixed-effect linear regression, the timepoint remained significant (*p* < 0.001), though there was a significant interaction between the timepoint and chemical class on the ADUR_DDD_, indicating changes in preferred classes. Table 12 displays the coefficients for this mixed-effects model in each province evaluated at both times.

**Table 11:** The mean parenteral ADUR_DCD_ (DCD/100 adult cow-years) and ADUR_DDD_ (DDD/100 adult cow-years) for each region at each time point.

	2007–2008		2018–2020	
Region	ADUR_DCD_	ADUR_DDD_	ADUR_DCD_	ADUR_DDD_
Fraser valley (British Columbia)	N/A	N/A	126	607
Calgary-southeast (Alberta)	510	1,660	248	953
London-Middlesex (Ontario)	313	1,262	227	1,141
Maritime provinces	407	1,355	284	951
Québec	412	1,625	228	822

**Table 12:** Mixed-effects linear model for natural logarithm of ADUR_DDD_ for each chemical class in provinces observed at each time point, with the interaction between the chemical class and time.

		Alberta		Ontario		Québec		Nova Scotia	
		Coeff.	95%CI	Coeff.	95%CI	Coeff.	95%CI	Coeff.	95%CI
Time	2007–2008	Ref.		Ref.		Ref.		Ref.	
	2018–2020	**−1.20**	−2.01, −0.39	−0.30	−1.03, 0.44	**−1.10**	−1.59, −0.60	**−1.11**	−2.16, −0.06
Class	Penicillin	Ref.		Ref.		Ref.		Ref.	
	Aminoglycoside	**−2.01**	−2.81, −1.21	**−1.91**	−2.61, −1.21	**−2.45**	−3.03, −1.87	**−2.69**	−3.60, −1.78
	Amphenicol	**−3.30**	−4.27, −2.32	**−3.39**	−4.36, −2.41	**−3.40**	−4.32, −2.48	**−3.75**	−5.23, −2.26
	First-generation cephalosporin	**−3.27**	−4.10, −2.44	**−1.39**	−2.12, −0.67	**−2.65**	−3.25, −2.05	**−1.76**	−2.67, −0.85
	Fluoroquinolone	**−3.18**	−4.99, −1.38	**−3.71**	−5.59, −1.83	**−4.65**	−6.26, −3.04	−2.62	−5.4, 0.15
	Lincosamide	**−3.03**	−3.92, −2.14	**−3.09**	−3.89, −2.3	**−3.65**	−4.31, −2.98	**−4.82**	−6.01, −3.62
	Macrolide	−0.98	−2.19, 0.24	**−1.35**	−2.32, −0.37	**−3.17**	−3.98, −2.37	**−3.85**	−5.53, −2.16
	Polymyxin	**−2.53**	−3.33, −1.73	**−2.42**	−3.12, −1.72	**−2.96**	−3.54, −2.38	**−3.2**	−4.11, −2.29
	TMS	**−2.45**	−3.28, −1.62	**−2.69**	−3.47, −1.92	**−3.23**	−3.82, −2.63	**−3.26**	−4.25, −2.26
	Tetracycline	**−2.35**	−3.18, −1.52	**−1.74**	−2.55, −0.92	**−3.01**	−3.64, −2.38	**−3.25**	−4.52, −1.99
	Third-generation cephalosporin	**−2.30**	−3.13, −1.47	**−1.79**	−2.47, −1.11	**−3.35**	−3.96, −2.74	**−2.21**	−3.12, −1.30
Interaction	Penicillin	Ref.		Ref.		Ref.		Ref.	
	Aminoglycoside	0.30	−0.72, 1.32	−0.47	−1.54, 0.59	0.62	−0.05, 1.28	0.92	−0.35, 2.20
	Amphenicol	1.47	0.28, 2.65	**1.76**	0.54, 2.99	0.77	−0.28, 1.82	**2.38**	0.37, 4.39
	First-generation cephalosporin	**3.23**	2.11, 4.35	0.48	−0.54, 1.49	**0.69**	0.01, 1.38	0.17	−1.14, 1.48
	Fluoroquinolone	0.71	−1.43, 2.86	N/A	N/A	1.63	−1.11, 4.37	N/A	N/A
	Lincosamide	0.99	−0.41, 2.39	−0.12	−1.51, 1.26	**1.57**	0.83, 2.31	**3.15**	1.35, 4.94
	Macrolide	−0.69	−2.19, 0.81	0.74	−0.64, 2.12	**1.45**	0.52, 2.38	**3.80**	1.55, 6.04
	Polymyxin	0.33	−0.69, 1.35	−0.5	−1.55, 0.55	0.62	−0.05, 1.28	0.93	−0.36, 2.22
	TMS	0.01	−1.05, 1.07	0.44	−0.59, 1.48	**0.72**	0.04, 1.4	0.53	−0.8, 1.87
	Tetracycline	0.60	−0.51, 1.71	−0.55	−1.72, 0.62	0.71	−0.04, 1.46	1.28	−0.50, 3.07
	Third-generation cephalosporin	**1.94**	0.91, 2.97	0.94	−0.01, 1.89	**1.71**	1.03, 2.4	1.17	−0.08, 2.42
Constant		**6.23**	5.59, 6.88	**5.74**	5.2, 6.27	**6.52**	6.08, 6.95	**6.02**	5.25, 6.79

Bolding indicates significance. Fluoroquinolones were not used in 2007–2008 in Ontario and Nova Scotia.

## 4. Discussion

Overall, antimicrobial use decreased substantially and pervasively from 2007 to 2008 (10, 11). In multivariable models, the time point the data were collected at was the most important explanatory variable for the reduction in ADUR_DDD_. The only noteworthy increase was in third-generation cephalosporins, which is attributable to the introduction of IMM ceftiofur products to the industry (19). This drop in overall AMU is likely due to a combination of the transition to selective dry cow therapy from blanket protocols, changes in producer priorities, industry group standards, veterinary guidelines, government intervention, and consumer preferences.

There are several differences in the context of AMU in the Canadian dairy industry between the previous effort by Saini et al. (10) in 2007–2008 and more recent projects (10). The primary difference was legislative changes, the most prominent being federal regulations that went into effect in August of 2018, which indicated all antimicrobials for use in animals now require a veterinary prescription (20). This change presented a layer of friction and oversight in antimicrobial acquisition, as it became less convenient for farms to procure antimicrobials and necessitated veterinary approval. Further regulations came into effect in Québec in 2019, directed at reducing the use of Category I. Millar et al. (18) found that these regulations were extremely effective and may provide a further intervention to address ceftiofur usage (18).

Another change was the introduction of new drugs and the removal of others from 2007 until 2020. A polymyxin combination (Special Formula 17,900-Forte, Zoetis Canada, Kirkland, QC, Canada) was widely used as an IMM treatment or, extralabelly, for keratoconjunctivitis. This product had three medically important antimicrobial agents. However, this product went off the market in Canada in July 2021, after years of supply issues coinciding with the later GCAs of this study. As time goes on, change in the market necessitates new decisions and protocols at the farm and veterinary clinic levels. Therefore, producers may have made a switch to cephalosporin products after Special Formula became unavailable.

Other AMU quantification studies have been conducted in the dairy industries of different parts of the world. Using a GCA, AMU was comparable in Flanders (759 DDD per 100 cow-yr) than the 623 d per 100 animal-yr reported in this study (21). More recently, using treatment records in large Wisconsin dairy farms was reported to be 628 DDD per 100 animal-yr (6), similar to our study. Notably, these studies were performed in different populations and used different data collection methods, so comparisons are made with caution.

In Saini et al. (10), IMM penicillin use was driven by Special Formula Forte-17,900. This udder injector contained several active ingredients, including the antimicrobials penicillin G procaine, polymyxin B sulfate, and dihydrostreptomycin. Polymyxins and aminoglycosides were rarely, if ever, used outside of this product. This formulation led to a high correlation between polymyxin use and aminoglycoside use, as well as a moderate correlation between polymyxin and penicillin use. It is worth noting that many medications used to treat calf diarrhea contain many medically important antimicrobial AIs, primarily sulfonamides, but are infrequently used, so they present less of a challenge to AMU quantification.

Systemic (intramuscular, intravenous, or subcutaneous) use of antimicrobials occurred on every farm evaluated in 2019–2020. Intramammary administration was nearly as common, but not used by every farm (101/107 farms). IMM use accounted for two-thirds of total AMU. This highlights that bacterial mastitis is still the most important infectious condition in the Canadian dairy industry (22).

In the 2019–2020 data set, farms with higher milk yields had a higher use of third-generation cephalosporins. This may be due to aggressive treatment leading to faster resolution of clinical mastitis cases with less of a lasting effect on milk production (23). This explanation may also apply for the negative correlation between the ADUR_DDD_ of third-generation cephalosporins and SCC. Farms that are aggressively trying to reduce their SCC or maintain a low SCC may establish more aggressive protocols for treating and preventing mastitis during lactation and at dry-off.

The negative correlation between herd size and total ADUR_DDD_ is unclear. This relationship indicates that larger herds use lower volumes of antimicrobials per animal per year than smaller herds. This could simply be due to expired products being more common on smaller farms because the drug turnover is lower, causing an outsized impact when looking at discarded products. It could also be due to increased resources and specialization in larger herds. It appears unlikely that an AMU reduction strategy should include increasing herd size, but, rather, AMU quantification programs should choose population correction units carefully and provide benchmarking to similar farms on multiple dimensions.

One problem with GCAs for quantifying AMU is rounding partly used quantities of large format drugs. For example, one farm in this study used half of a bag of a chlortetracycline feed premix during the observation period. This half bag accounted for 15,278 DDD. The farm with the second-highest usage had 5,441 DDD for all drugs on the farm. Since the fraction of the bag remaining was estimated visually, the fraction can be off by what would amount to the total use of another high-AMU farm. To account for this, an option would be to exclude this drug from this farm’s metrics. However, this fails to account for the large amount of chlortetracycline used by this farm, and we believe this use should be accounted for. The high DDD from this farm and others like it is due to the outsized contribution of feed additives to the DDD count. This farm had an ADUR_DCD_ of 331 DCD/100 animal-yr, which is high relative to the average of farms in that collection effort of 117 DCD/100 animal-yr, but much lower than the maximum of 499 DCD/100 animal-yr.

Another potential measurement issue could be an insufficient length of observation to encapsulate usage patterns. A GCA measures AMU through discreet, discarded items. This study intended to leave the garbage cans for 6 months, which is shorter than what has been done in other projects using GCAs (10, 21). This length may not reflect year-round AMU, due to seasonality of infection pressure and incidence of conditions like mastitis. However, most were left far longer, some over a year, which is more in line with standard efforts. Once a GCA lasts longer than a year, it should provide a valid snapshot of use, with reduced impact of seasonality.

When quantifying AMU in any production system, an important question to consider is how long the observation period must be to determine the ADUR properly. Veterinary dispensing records have the advantages of providing sales dates and containing much longer periods of retrospective data much of the time. However, using veterinary dispensing records requires building the infrastructure to process the data and relationships with producers and veterinary clinics to provide the dispensing data. These data could determine the dispersion in ADUR as a function of time to determine the necessary sample period for a given effect size. The implicit assumption in most of this research is that these estimates are consistent over reasonable periods, and future research should evaluate this assumption by measuring AMU on farms over extended periods.

## 5. Conclusion

There was a significant and ubiquitous drop in AMU across the Canadian dairy industry since 2008. The notable exception being third-generation cephalosporins, which is mostly attributable to the introduction of intramammary ceftiofur products. Intramammary administration accounts for most AMU in the Canadian dairy industry. The finding that milk production and SCC are associated with AMU provides opportunities for the industry to improve production and antimicrobial stewardship together. The Canadian dairy industry is well-positioned to continue improving antimicrobial stewardship.

## Data availability statement

The original contributions presented in the study are included in the article/Supplementary material, further inquiries can be directed to the corresponding author.

## Ethics statement

Ethics review and approval/written informed consent was not required as per local legislation and institutional requirements.

## Author contributions

LW developed the data processing system, analyzed the data, and wrote and edited the manuscript. LH, DL, DRi, and JS planned the data collection, set up logistics, helped with analysis, and edited the manuscript. DK, DRe, SD, HB, and J-PR designed the sampling, set up logistics, and edited the manuscript. All authors contributed to the article and approved the submitted version.

## Funding

This research was funded by the Dairy Research Cluster 3 (Dairy Farmers of Canada and Agriculture and Agri-Food Canada) under the Canadian Agricultural Partnership AgriScience Program and the Public Health Agency of Canada.

## Publisher’s note

All claims expressed in this article are solely those of the authors and do not necessarily represent those of their affiliated organizations, or those of the publisher, the editors and the reviewers. Any product that may be evaluated in this article, or claim that may be made by its manufacturer, is not guaranteed or endorsed by the publisher.

## Conflict of interest

The authors declare that the research was conducted in the absence of any commercial or financial relationships that could be construed as a potential conflict of interest.

## Supplementary material

The Supplementary material for this article can be found online at: https://www.frontiersin.org/articles/10.3389/fvets.2023.1185628/full#supplementary-material

## References

[1] WHO–World Health Organization (2020). Antibiotic resistance. Available at: https://www.who.int/news-room/fact-sheets/detail/antibiotic-resistance#:~:text=Key%20facts,animals%20is%20accelerating%20the%20process (Accessed August 3, 2022).

[2] Government of Canada (2020). Antimicrobial resistance and animals – actions. Available at: https://www.canada.ca/en/public-health/services/antibiotic-antimicrobial-resistance/animals/actions.html (Accessed May 30, 2021).

[3] IACG – International Coordination Group on Antimicrobial Resistance (2018). Surveillance and monitoring for antimicrobial use and resistance. Available at: https://www.who.int/docs/default-source/antimicrobial-resistance/amr-gcp-tjs/iacg-surveillance-and-monitoring-for-amu-and-amr-110618.pdf?sfvrsn=8a07c166_4 (Accessed June 22, 2022).

[4] Sanders P Vanderhaeghen W Fertner M Fuchs K Obritzhauser W Agunos A. Monitoring of farm-level antimicrobial use to guide stewardship: overview of existing systems and analysis of key components and processes. Front Vet Sci. (2020) 7:540. doi: 10.3389/fvets.2020.00540, PMID: 33195490 PMC7475698

[5] Pucken V-B Bodmer M Lovis B Pont J Savioli G Sousa FM. Antimicrobial consumption: comparison of three different data collection methods. Prev Vet Med. (2021) 186:105221. doi: 10.1016/j.prevetmed.2020.105221, PMID: 33310589

[6] de Campos J Kates A Steinberger A Sethi A Suen G Shutske J. Quantification of antimicrobial usage in adult cows and preweaned calves on 40 large Wisconsin dairy farms using dose-based and mass-based metrics. J Dairy Sci. (2021) 104:4727–45. doi: 10.3168/jds.2020-19315, PMID: 33551167

[7] Menéndez González S Steiner A Gassner B Regula G. Antimicrobial use in Swiss dairy farms: quantification and evaluation of data quality. Prev Vet Med. (2010) 95:50–63. doi: 10.1016/j.prevetmed.2010.03.004, PMID: 20381180

[8] Nobrega DB De Buck J Naqvi SA Gang L Naushad S Saini V. Comparison of treatment records and inventory of empty drug containers to quantify antimicrobial usage in dairy herds. J Dairy Sci. (2017) 100:9736–45. doi: 10.3168/jds.2017-13116, PMID: 28987586

[9] Redding LE Bender J Baker L. Quantification of antibiotic use on dairy farms in Pennsylvania. J Dairy Sci. (2019) 102:1494–507. doi: 10.3168/jds.2018-15224, PMID: 30594359

[10] Saini V McClure JT Léger D Dufour S Sheldon AG Scholl DT. Antimicrobial use on Canadian dairy farms. J Dairy Sci. (2012) 95:1209–21. doi: 10.3168/jds.2011-4527 22365205

[11] Lardé H Francoz D Roy J-P Massé J Archambault M Paradis M-È. Comparison of quantification methods to estimate farm-level usage of antimicrobials other than in medicated feed in dairy farms from Québec, Canada. Microorgansisms. (2021) 9:1106. doi: 10.3390/microorganisms9051106, PMID: 34576729 PMC8471653

[12] ESVAC – European Medicines Agency European Surveillance of Veterinary Antimicrobial Consumption (2015). Principles on assignment of defined daily dose for animals (DDDvet) and defined course dose for animals (DCDvet). Available at: https://www.ema.europa.eu/en/documents/scientific-guideline/principles-assignment-defined-daily-dose-animals-dddvet-defined-course-dose-animals-dcdvet_en.pdf (Accessed May 30, 2021).

[13] Lardé H Dufour S Archamault M Leger D Loest D Roy J-P. Assignment of Canadian defined daily doses and Canadian defined course doses for quantification of antimicrobial usage in cattle. Front Vet Sci. (2020) 7:10. doi: 10.3389/fvets.2020.00010, PMID: 32083099 PMC7001643

[14] Fonseca M Heider LC Léger D McClure JT Rizzo D Dufour S. Canadian dairy network for antimicrobial stewardship and resistance (CaDNetASR): an on-farm surveillance system. Front Vet Sci. (2022) 8:799622. doi: 10.3389/fvets.2021.799622, PMID: 35097047 PMC8790291

[15] Government of Canada (2021). Dairy barns by type. Available at: https://agriculture.canada.ca/en/sector/animal-industry/canadian-dairy-information-centre/statistics-market-information/farm-statistics/barns-type (Accessed May 17, 2023)

[16] NFACC – National Farm Animal Care Council (2023). Code of practice for the care and handling of dairy cattle. Available at: https://www.nfacc.ca/codes-of-practice/dairy-cattle/code2023#section2 (Accessed May 17, 2023).

[17] Health Canada (2009). Categorization of antimicrobial drugs based on importance in human medicine. Available at: https://www.canada.ca/en/health-canada/services/drugs-health-products/veterinary-drugs/antimicrobial-resistance/categorization-antimicrobial-drugs-based-importance-human-medicine.html (Accessed May 30, 2021).

[18] Millar N Aenishaenslin C Lardé H Roy J-P Fourichon C Francoz D. Evidence of a decrease in sales of antimicrobials of very high importance for humans in dairy herds after a new regulation restricting their use in Quebec. Canada Zoonoses Public Health. (2021) 69:370–81. doi: 10.1111/zph.12929, PMID: 35199952

[19] Health Canada – Government of Canada (2022). Product information – Spectramast LC. Available at: https://health-products.canada.ca/dpd-bdpp/dispatch-repartition.do;jsessionid=476D2DE49EA59BCE6936C4D95295845D (Accessed September 30, 2022).

[20] Health Canada (2021). Responsible use of medically important antimicrobials in animals. Available at: https://www.canada.ca/en/public-health/services/antibiotic-antimicrobial-resistance/animals/actions/responsible-use-antimicrobials.html (Accessed March 7, 2023).

[21] Stevens M Piepers S Supre K Dewulf J De Vliegher S. Quantification of antimicrobial consumption in adult cattle on dairy herds in Flanders, Belgium, and associations with udder health, milk quality, and production performance. J Dairy Sci. (2016) 99:2118–30. doi: 10.3168/jds.2015-10199, PMID: 26778315

[22] Jamali H Barkema HW Jacques M Lavallée-Bourget E-M Malouin F Saini V. Invited review: incidence, risk factors, and effects of clinical mastitis recurrence in dairy cows. J Dairy Sci. (2018) 101:4729–46. doi: 10.3168/jds.2017-13730, PMID: 29525302

[23] Adriaens I Van Den Brulle I Geerinckx K D’Anvers L De Vliegher S Aernouts B. Milk losses linked to mastitis treatments at dairy farms with automatic milking systems. Prev Vet Med. (2021) 194:105420. doi: 10.1016/j.prevetmed.2021.105420 34274863

