# Supplementary material for: Quantifying antimicrobial use on Canadian dairy farms using garbage can audits
Source: Front Vet Sci. 2023 Jun 30;10:1185628. doi: 10.3389/fvets.2023.1185628 (PMC10347401; doi:10.3389/fvets.2023.1185628)
Supplement: Supplementary file 1 [file Table_1.pdf]

Supplementary material.

Table S1. Univariable mixed-effects linear regression between the number of animals on a farm versus the natural logarithm of the $ADUR_{DDD}$.

ln(ADURddd)	Coef.	Std. Err.	z	P>z	[95% Conf. Interval]	
Herd Size	-0.001195	0.0002912	-4.1	0	-0.0017658	-0.0006242
Constant	3.536986	0.1255314	28.18	0	3.290949	3.783023

Random-effects Parameters	Estimate	Std. Err.	[95% Conf. Interval]	
Farn: Identity				
var(Constant)	0.110444	0.070399	0.031664	0.385224
var(Residual)	2.616893	0.147296	2.343553	2.922113

Table S2. Univariable mixed-effects linear regression between SCC log score of a farm versus the natural logarithm of the $ADUR_{DDD}$.

ln(ADURddd)	Coef.	Std. Err.	z	P>z	[95% Conf. Interval]	
SCC Log Score	-0.008058	0.209596	-0.04	1	-0.4188581	0.4027431
Constant	3.160719	0.4794859	6.59	0	2.220944	4.100494

Random-effects Parameters	Estimate	Std. Err.	[95% Conf. Interval]	
Farm: Identity				
var(Constant)	0.189163	0.082767	0.080242	0.445937
var(Residual)	2.520366	0.151366	2.24049	2.835203

Table S3. Univariable mixed-effects linear regression between the 305-day milk production a farm versus the natural logarithm of the ADUR$_{DDD}$.

ln(ADURddd)	Coef.	Std. Err.	z	P>z	[95% Conf. Interval]	
305-d milk production	2.34E-05	5.63E-05	0.42	0.678	-0.0000869	0.000134
Constant	2.871697	0.632211	4.54	0	1.632586	4.110808

Random-effects Parameters	Estimate	Std. Err.	[95% Conf. Interval]	
Farm: Identity				
var(Constant)	0.19656	0.083272	0.085682	0.450923
var(Residual)	2.544114	0.151113	2.264528	2.858218

Table S4. Mixed effects linear regression of the natural logarithm of the ADUR$_{DDD}$ versus the chemical class of the antimicrobial, with aminocoumarins as reference, and the number of animals in the herd.

ln(ADURddd)	Coef.	Std. Err.	z	P>z	[95% Conf. Interval]	
Chemical Class						
Aminoglycoside	-0.52611	0.224076	-2.35	0.019	-0.96529	-0.08693
Amphenicol	-0.33021	0.227231	-1.45	0.146	-0.77557	0.115159
1st Gen. Cephalosporin	1.124124	0.229698	4.89	0	0.673924	1.574323
Fluoroquinolone	-1.4414	0.420722	-3.43	0.001	-2.266	-0.6168
Lincosamide	-1.00781	0.327755	-3.07	0.002	-1.6502	-0.36542
Macrolide	0.366874	0.280746	1.31	0.191	-0.18338	0.917126
Penicillin	1.436791	0.204898	7.01	0	1.035199	1.838383
Polymyxin	-1.03693	0.224076	-4.63	0	-1.47611	-0.59775
TMS	-0.74249	0.213889	-3.47	0.001	-1.1617	-0.32327
Tetracycline	-0.65756	0.258582	-2.54	0.011	-1.16437	-0.15075
3rd Gen. Cephalosporin	0.882537	0.205477	4.3	0	0.47981	1.285265
Herd Size	-0.00101	0.000295	-3.44	0.001	-0.00159	-0.00044
Constant	3.365487	0.195574	17.21	0	2.982169	3.748805

Random-effects Parameters	Estimate	Std. Err.	[95% Conf. Interval]	
Farm: Identity				
var(Constant)	0.248315	0.070509	0.142333	0.433214
var(Residual)	1.715718	0.09661	1.536441	1.915913

Table S5. Mixed effects linear regression of the natural logarithm of the ADURDDD versus the chemical class of the antimicrobial, with aminocoumarins as reference, and the logarithm of the somatic cell count.

ln(ADURddd)	Coef.	Std. Err.	z	P>z	[95% Conf. Interval]	
Chemical Class						
Aminoglycoside	-0.50002	0.234069	-2.14	0.033	-0.95878	-0.04125
Amphenicol	-0.45835	0.240035	-1.91	0.056	-0.9288	0.012113
1st Gen. Cephalosporin	1.108174	0.23779	4.66	0	0.642114	1.574235
Fluoroquinolone	-1.85374	0.451356	-4.11	0	-2.73838	-0.9691
Lincosamide	-0.95622	0.346131	-2.76	0.006	-1.63462	-0.27781
Macrolide	0.157016	0.301346	0.52	0.602	-0.43361	0.747645
Penicillin	1.310868	0.214245	6.12	0	0.890956	1.73078
Polymyxin	-1.01084	0.234069	-4.32	0	-1.46961	-0.55208
TMS	-0.90418	0.224728	-4.02	0	-1.34464	-0.46372
Tetracycline	-0.69031	0.277724	-2.49	0.013	-1.23464	-0.14598
3rd Gen. Cephalosporin	0.781409	0.214962	3.64	0	0.36009	1.202727
SCC Log Score	0.053481	0.206036	0.26	0.795	-0.35034	0.457305
Constant	2.976737	0.504534	5.9	0	1.987869	3.965605

Random-effects Parameters	Estimate	Std. Err.	[95% Conf. Interval]	
Farm: Identity				
var(Constant)	0.29675	0.079741	0.17525	0.502483
var(Residual)	1.652451	0.099385	1.468704	1.859188

Table S6. Mixed effects linear regression of the natural logarithm of the $ADUR_{DDD}$ versus the chemical class of the antimicrobial, with aminocoumarins as reference, and the average milk output, in kilograms, per 305-days of lactation.

ln(ADURddd)	Coef.	Std. Err.	z	P>z	[95% Conf. Interval]	
Chemical Class						
Aminoglycoside	-0.50412	0.231357	-2.18	0.029	-0.95757	-0.05067
Amphenicol	-0.42109	0.237907	-1.77	0.077	-0.88737	0.045204
1st Gen. Cephalosporin	1.121823	0.236889	4.74	0	0.65753	1.586116
Fluoroquinolone	-1.84305	0.452425	-4.07	0	-2.72978	-0.95631
Lincosamide	-0.94023	0.346555	-2.71	0.007	-1.61947	-0.261
Macrolide	0.124951	0.297228	0.42	0.674	-0.45761	0.707507
Penicillin	1.332002	0.212045	6.28	0	0.916402	1.747602
Polymyxin	-1.01494	0.231357	-4.39	0	-1.46839	-0.56149
TMS	-0.92244	0.222728	-4.14	0	-1.35897	-0.4859
Tetracycline	-0.60495	0.274887	-2.2	0.028	-1.14372	-0.06618
3rd Gen. Cephalosporin	0.81066	0.212744	3.81	0	0.393689	1.227631
305d Milk Output	2.84E-05	0.000055	0.52	0.606	-7.9E-05	0.000136
Constant	2.760678	0.635172	4.35	0	1.515764	4.005591

Random-effects Parameters	Estimate	Std. Err.	[95% Conf. Interval]	
Farm: Identity				
var(Constant)	0.303744	0.080035	0.181226	0.509092
var(Residual)	1.667904	0.099207	1.484368	1.874133

Table S7. Linear regression of the natural logarithm $ADUR_{DDD}$ of all parenteral antimicrobials for all regions assessed with Alberta as the reference.

ln(ADURddd)	Coef.	Std. Err.	t	P>t	[95% Conf. Interval]	
Province						
British Columbia	-0.41604	0.200796	-2.07	0.041	-0.81431	-0.01776
Nova Scotia	-0.04123	0.21405	-0.19	0.848	-0.46579	0.38334
Ontario	0.239474	0.189901	1.26	0.21	-0.13719	0.616143
Constant	6.118711	0.135377	45.2	0	5.850192	6.387231

Table S8. Linear regression of the natural logarithm $ADUR_{DCD}$ of all parenteral antimicrobials for all regions assessed with Alberta as the reference.

ln(ADURdcd)	Coef.	Std. Err.	t	P>t	[95% Conf. Interval]	
Province						
British Columbia	-0.68239	0.204726	-3.33	0.001	-1.08846	-0.27632
Nova Scotia	-0.2055	0.218239	-0.94	0.349	-0.63838	0.227373
Ontario	-0.17978	0.193618	-0.93	0.355	-0.56382	0.204258
Constant	4.750599	0.138027	34.42	0	4.476824	5.024374

Table S9. Linear regression of the natural logarithm $ADUR_{DDD}$ of all systemically administered antimicrobials for all regions assessed with Alberta as the reference.

ln(ADURddd)	Coef.	Std. Err.	t	P>t	[95% Conf. Interval]	
Province						
British Columbia	-0.13055	0.272305	-0.48	0.633	-0.67066	0.409568
Nova Scotia	-0.3352	0.290278	-1.15	0.251	-0.91097	0.24056
Ontario	0.212964	0.25753	0.83	0.41	-0.29784	0.723774
Constant	4.580709	0.183588	24.95	0	4.216564	4.944854

Table S10. Linear regression of the natural logarithm $ADUR_{DDD}$ of all systemically administered antimicrobials for all regions assessed with Alberta as the reference.

ln(ADURdcd)	Coef.	Std. Err.	t	P>t	[95% Conf. Interval]	
Province						
British Columbia	-0.16089	0.262707	-0.61	0.542	-0.68197	0.36019
Nova Scotia	-0.40295	0.280046	-1.44	0.153	-0.95842	0.152517
Ontario	0.162993	0.248453	0.66	0.513	-0.32981	0.655798
Constant	3.134867	0.177117	17.7	0	2.783557	3.486178

Table S11. Linear regression of the natural logarithm $ADUR_{DDD}$ of all intramammary antimicrobials for all regions assessed with Alberta as the reference.

ln(ADURddd)	Coef.	Std. Err.	t	P>t	[95% Conf. Interval]	
Province						
British Columbia	-0.46835	0.320456	-1.46	0.147	-1.10437	0.167664
Nova Scotia	-0.01758	0.337271	-0.05	0.959	-0.68696	0.651813
Ontario	0.26361	0.305219	0.86	0.39	-0.34216	0.869384
Constant	5.609447	0.217707	25.77	0	5.177359	6.041536

Table S12. Linear regression of the natural logarithm $ADUR_{DCD}$ of all intramammary antimicrobials for all regions assessed with Alberta as the reference.

ln(ADURdcd)	Coef.	Std. Err.	t	P>t	[95% Conf. Interval]	
Province						
British Columbia	-0.89462	0.290361	-3.08	0.003	-1.4709	-0.31833
Nova Scotia	-0.17341	0.305597	-0.57	0.572	-0.77994	0.433112
Ontario	-0.2392	0.276555	-0.86	0.389	-0.78808	0.309685
Constant	4.331826	0.197262	21.96	0	3.940316	4.723336

Table S13. Mixed effect linear regression of the $ADUR_{DDD}$ for each class of antimicrobial from Saini et al. (2012) to Lardé et al. (2021) and Fonseca et al. (2022), with aminocoumarins and Saini as the reference.

ln(ADURddd)	Coef.	Std. Err.	z	P>z	[95% Conf. Interval]	
Timepoint						
2018-2020	-1.07393	0.189165	-5.68	0	-1.44469	-0.70317
Chemical class						
Aminoglyco.	-1.26916	0.198749	-6.39	0	-1.6587	-0.87962
Amphenicols	-2.45165	0.28319	-8.66	0	-3.00669	-1.89661
F.G. Ceph.	-1.24832	0.204671	-6.1	0	-1.64946	-0.84717
Fluoroquin.	-2.80453	0.682211	-4.11	0	-4.14164	-1.46742
Lincosamides	-2.51864	0.229581	-10.97	0	-2.96861	-2.06867
Macrolides	-1.25393	0.291466	-4.3	0	-1.82519	-0.68267
Penicillins	0.974492	0.196604	4.96	0	0.589156	1.359828
Polymyxins	-1.78123	0.198749	-8.96	0	-2.17077	-1.39169
Sulfonamides	-0.60675	0.315796	-1.92	0.055	-1.2257	0.012203
TMS	-1.91169	0.211397	-9.04	0	-2.32602	-1.49736
Tetracyclines	-1.51798	0.224683	-6.76	0	-1.95835	-1.07761
T.G. Ceph.	-1.44728	0.201809	-7.17	0	-1.84282	-1.05174
Timepoint#Chemical class						
2018-2020#Aminoglyco.	0.575779	0.247389	2.33	0.02	0.090905	1.060653
2018-2020#Amphenicols	1.603582	0.334021	4.8	0	0.948914	2.258251
2018-2020#F.G. Ceph.	1.166283	0.256453	4.55	0	0.663644	1.668922
2018-2020#Fluoroquin.	0.891423	0.78354	1.14	0.255	-0.64429	2.427133
2018-2020#Lincosamides	1.584051	0.285046	5.56	0	1.025372	2.14273
2018-2020#Macrolides	0.988521	0.353181	2.8	0.005	0.296299	1.680743
2018-2020#Penicillins	0.130196	0.239823	0.54	0.587	-0.33985	0.600241
2018-2020#Polymyxins	0.542959	0.248035	2.19	0.029	0.05682	1.029099
2018-2020#TMS	0.680267	0.256548	2.65	0.008	0.177441	1.183092
2018-2020#Tetracyclines	0.805846	0.28144	2.86	0.004	0.254234	1.357459
2018-2020#T.G. Ceph.	1.464582	0.24511	5.98	0	0.984174	1.944989
Constant	5.16118	0.153607	33.6	0	4.860117	5.462243

Table S14. Univariable regression of the natural logarithm of the $ADUR_{DCD}$ versus the time point when the studies were conducted

ln(ADURdcd)	Coef.	Std. Err.	z	P>z	[95% Con	Interval]
Time point	-0.3531	0.095224	-3.71	0	-0.53973	-0.16646
Constant	3.30958	0.166518	19.88	0	2.983211	3.635948
